# Local Absence of Secondary Structure Permits Translation of mRNAs that Lack Ribosome-Binding Sites

**DOI:** 10.1371/journal.pgen.1002155

**Published:** 2011-06-23

**Authors:** Lars B. Scharff, Liam Childs, Dirk Walther, Ralph Bock

**Affiliations:** Max-Planck-Institut für Molekulare Pflanzenphysiologie, Potsdam-Golm, Germany; Universidad de Sevilla, Spain

## Abstract

The initiation of translation is a fundamental and highly regulated process in gene expression. Translation initiation in prokaryotic systems usually requires interaction between the ribosome and an mRNA sequence upstream of the initiation codon, the so-called ribosome-binding site (Shine-Dalgarno sequence). However, a large number of genes do not possess Shine-Dalgarno sequences, and it is unknown how start codon recognition occurs in these mRNAs. We have performed genome-wide searches in various groups of prokaryotes in order to identify sequence elements and/or RNA secondary structural motifs that could mediate translation initiation in mRNAs lacking Shine-Dalgarno sequences. We find that mRNAs without a Shine-Dalgarno sequence are generally less structured in their translation initiation region and show a minimum of mRNA folding at the start codon. Using reporter gene constructs in bacteria, we also provide experimental support for local RNA unfoldedness determining start codon recognition in Shine-Dalgarno–independent translation. Consistent with this, we show that AUG start codons reside in single-stranded regions, whereas internal AUG codons are usually in structured regions of the mRNA. Taken together, our bioinformatics analyses and experimental data suggest that local absence of RNA secondary structure is necessary and sufficient to initiate Shine-Dalgarno–independent translation. Thus, our results provide a plausible mechanism for how the correct translation initiation site is recognized in the absence of a ribosome-binding site.

## Author Summary

Protein biosynthesis (translation) is a highly regulated process in gene expression. In all organisms, initiation of translation depends on molecular recognition of the messenger RNA by ribosomes. In prokaryotes (bacteria, mitochondria, and chloroplasts), this recognition is mediated by a specific sequence motif in the 5′ untranslated region of the mRNA, called “ribosome-binding site” or “Shine-Dalgarno sequence.” However, many messenger RNAs lack Shine-Dalgarno sequences, and it is currently unknown how the correct translation initiation site is recognized in these mRNAs. Here, we provide insights into the mechanism of translation initiation in the absence of a ribosome-binding site. We have performed genome-wide searches for Shine-Dalgarno–independent translation in bacterial and organellar genomes and report that a large fraction of transcripts is translated in a Shine-Dalgarno–independent manner in all prokaryotic systems. We find that Shine-Dalgarno–independent translation initiation is strongly correlated with the presence of a local minimum in RNA secondary structure around the translational start codon. The significance of RNA unfoldedness as the key determinant of start codon recognition in Shine-Dalgarno–independent translation initiation was confirmed experimentally by employing reporter gene fusions in the bacterium *Escherichia coli*. In conclusion, our work suggests an intriguing mechanism for translation initiation on mRNAs that lack a ribosome-binding site.

## Introduction

Shine-Dalgarno (SD) sequences reside in the 5′ untranslated region (5′ UTR) of prokaryotic messenger RNAs and facilitate translation initiation. They act as ribosome-binding sites by recognizing a sequence motif at the 3′ end of the 16S ribosomal RNA in the 30S ribosomal subunit (referred to as anti-Shine-Dalgarno sequence, ASD) via complementary base pairing [1], [2]. The SD-ASD interaction is conserved across the prokaryotic kingdom and has even been retained in some cell organelles that evolved from prokaryotes more than a billion years ago. For example, tobacco plastids (chloroplasts) and the γ-proteobacterium *Escherichia coli* have identical ASD sequences in the 3′ end of their 16S ribosomal RNAs (5′ TGGATCACCTCCTT 3′; ASD motif underlined) and, therefore, plastid SD sequences can be recognized in *E. coli* and *vice versa* [3], [4]. The SD consensus sequence is GGAGG in both systems. For efficient translation initiation to occur, the SD sequence needs to be present in the appropriate distance upstream of the start codon. The conserved spacing determined for *E. coli* is 4-9 nucleotides.

Although the SD-dependent mechanism of translation initiation appears to be highly conserved among prokaryotes, it has long been known that an alternative mechanism of translation initiation must exist that is independent of the presence of a consensus SD sequence [5], [6]. Even some highly translated mRNAs in bacteria lack a recognizable SD motif indicating that translation initiation in the absence of an SD sequence can occur at high efficiency [6]. More recently, comparative analyses in several prokaryotes have indicated that SD-independent translation is much more widespread than previously appreciated [7]. However, how efficient translation is possible in the absence of a functional SD sequence and how faithful start codon recognition occurs is largely unknown. Here we have sought to identify sequence elements and/or structural motifs at the mRNA level that are involved in SD-independent initiation of protein biosynthesis. While we find no evidence for alternative sequence motifs or secondary structural requirements, we have discovered that mRNAs lacking an SD sequence exhibit a pronounced minimum in mRNA secondary structure at the translational start codon, suggesting that start codon accessibility is the major factor in SD-independent translation initiation. Using reporter gene constructs in *E. coli*, we confirm experimentally that local RNA unfoldedness is necessary and sufficient for initiation of protein biosynthesis in the absence of an SD sequence.

## Results

### Widespread Shine-Dalgarno–Independent Translation in All Major Groups of Prokaryotes

**Figure 1:**
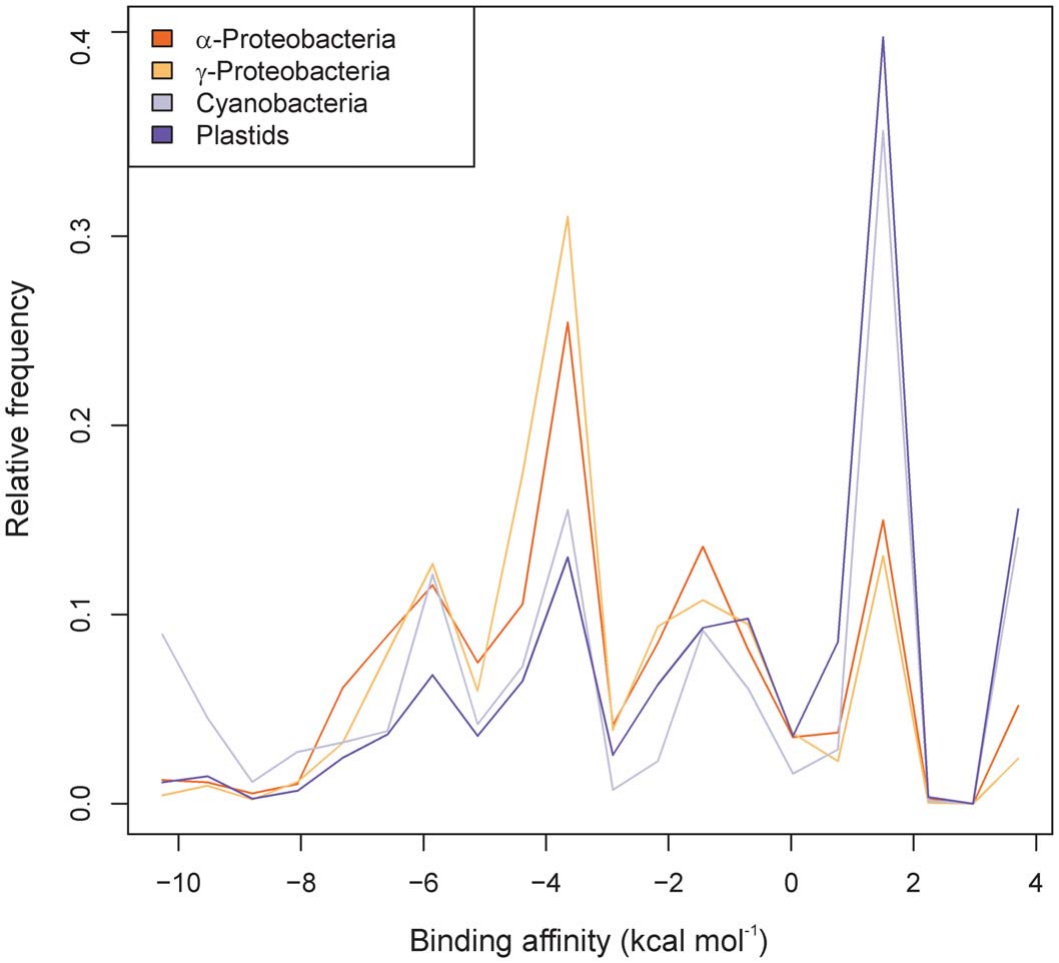
Genome-wide assessment of the binding affinities of the anti–Shine-Dalgarno sequence in the 16S rRNA to the 5′ UTRs of prokaryotic genes. The 5′ UTR sequences from -22 to -2 were used for the computations. Shown are the normalized distributions of hybridization energies of α-proteobacterial, γ-proteobacterical, cyanobacterial and plastid genes. Four major peaks are clearly distinguishable in all taxonomic groups at the positions -5.9, -3.6, -1.4 and +1.5 kcal mol^−1^. The peaks (from left to right) correspond to: (i) mRNAs with the SD sequence AGGAG, (ii) mRNAs with the SD sequence GAGG, AGGA or GGAG, (iii) mRNAs with short SD-like sequences (AGG, GAG or GGA), which may engage in SD-type interactions with the 3′ end of the 16S rRNA and (iv) mRNAs without SD sequences

When we searched 14,659 α-proteobacterial, 31,496 γ-proteobacterial, 6202 cyanobacterial and 11,238 plastid (chloroplast) gene sequences for the presence of the SD sequence motif, we found that 79.6% of α-proteobacterial genes, 84.2% of γ-proteobacterial genes, 49.6% of cyanobacterial genes and 60.1% of plastid genes have SD sequences (Figure 1; see Materials and Methods). This indicates a rather high number of mRNAs that are translated in an SD-independent manner in all prokaryotic systems and is in agreement with a recent study suggesting that the extent of SD-independent translation can greatly vary between different groups of prokaryotes [7]. Mitochondria represent the most extreme case in that the rRNA of the small subunit of the mitochondrial ribosome lacks the ASD motif and, consequently, SD sequences are generally absent from mitochondrial mRNAs [8].

### Absence of Conserved Sequence Motifs for Start Codon Recognition in SD–Independent Translation

The high proportion of genes without an SD sequence in all prokaryotic genomes (Figure 1) raises the question, how specific start codon recognition and efficient translation initiation occur in these mRNAs. It is conceivable that either sequence motifs in the 5′ untranslated region (5′ UTR) or secondary structural motifs (or a combination thereof) could act as recognition elements that direct the ribosomal 30S subunit to the initiation codon. This could either occur via direct interaction between the 30S ribosomal subunit and the 5′ UTR or, alternatively, be mediated by RNA-binding proteins. A paradigm for such a protein is ribosomal protein S1 (Rps1), which has been identified as a factor binding to AU-rich sequences upstream of the SD sequence and, in this way, can promote efficient translation initiation [9]–[11].

To identify possible alternative sequence motifs or structural motifs that could mediate start codon recognition and translation initiation, we first performed an unbiased search for conserved sequence motifs in the 5′ UTR employing the MEME algorithm [12]. The search was performed independently for one thousand randomly selected bacterial genes and one thousand randomly selected plastid genes, irrespective of the presence or absence of an SD sequence. As expected, the SD sequence was identified as a frequently occurring motif in both bacterial and plastid genes (see Materials and Methods for details). No other sequence motif occurred in more than 20 genes. To specifically search for possible sequence motifs that could promote SD-independent translation initiation, we filtered out all genes containing an SD sequence from the full set of 160,340 bacterial gene sequences and 10,420 plastid sequences. The remaining 71,626 bacterial and 6462 plastid genes without an SD sequence were searched for conserved sequence motifs using MEME. In bacteria, none of the detected motifs occurred in more than 11,000 (15%) of all 5′ UTRs lacking an SD sequence. In plastids, none of the detected motifs occurred in more than 750 (11%) of all 5′ UTRs lacking an SD sequence.

To confirm the absence of an SD-equivalent sequences motif in SD-independent translation, we performed an additional unbiased search for conserved sequence motifs in the 5′ UTR employing the Amadeus motif discovery platform [13]. Again, the SD sequence was identified as a frequently occurring motif in both bacterial and plastid genes. As expected, the SD sequence was identified as occurring more frequently than a shuffled background in 53.4% (p = 3.0 e^−108^) of all bacterial genes and 37.8% (p = 1.7 e^−97^) of all plastid genes (see Materials and Methods for details). No other motifs were detected as significantly enriched. After removal of all 5′UTRs with identified SD sequences, the remaining 5′ UTRs were re-analyzed. In the remaining plastid 5′ UTRs, two motifs were detected to occur significantly more often in 5′UTRs without SD sequences than a random or genomic background after correction for multiple testing. One motif (AAAGGT, p = 3.8 e^−16^) occurs in 21.9% of all the 5′UTRs and is likely to be a variant of the SD sequence. The other (TATAAT, p = 4.5 e^−16^) occurs in 29.3% of all plastid 5′ UTRs and is a canonical promoter element, the Pribnow (−10) box. In the remaining bacterial 5′ UTRs, one motif was detected to be overrepresented. This motif (AAAGGC, p = 6.0 e^−18^) occurs in 13.1% of all the 5′ UTRs and is also likely to be a variant of the SD sequence.

Taken together, these results indicate that there is no general sequence motif that replaces the SD sequence in SD-independent translation.

### Absence of Conserved Secondary Structural Motifs for Start Codon Recognition in SD–Independent Translation

We next considered the possibility that secondary structural motifs in the 5′ UTR (and/or the 5′ sequence of the coding region) promote start codon recognition in the absence of an SD sequence. To this end, we analyzed the same datasets of bacterial and plastid genes for the presence of conserved structural motifs. Using the RNAshapes algorithm [14], an abstract RNA structure that disregards stem length and loop size was obtained (see Materials an Methods for details). In bacteria, two motifs ([_[_[_[]]_]_] and [_[]_[[]_]_]]) had p-values of less than 0.01 and occurred in 21 and 5 of the 1000 random sequences, respectively, and in 1646 (1.0%) and 219 (0.13%) of the sequences of the entire set. (An opening bracket indicates that base pairing occurs between the represented region and the region of the matching closing bracket, an underscore indicates a stretch of unpaired nucleotides.) In plastids, eight motifs ([_[_[_[_[]_]]_]], [_[]_]_[_[]], [_[]]_[], [_[]], [[_[_[_[]_]_]_]_], [[]_], [_[_[_[_[]_]_]]_] and [_[_[_[]_]]_]) had p-values of less than 0.01 and occurred in 65 (0.62%), 138 (1.32%), 184 (1.77%), 1536 (14.74%), 16 (0.15%), 959 (9.20%), 13 (1.25%) and 128 (1.23%) of the sequences belonging to the entire set. Of note, the single hairpin motif ([]) occurs in 100% of all sequences. However, neither the position of the hairpin nor the size of the stem and the loop are conserved. Moreover, the probability that a hairpin will occur in random sequences based on the same nucleotide frequencies is very high and was observed in all tested random sequences. These data strongly argue against the presence of conserved secondary structural elements that could direct the ribosomal 30S subunit to the translation initiation codon, if no SD sequence is present in the 5′ UTR.

### Lack of mRNA Secondary Structure at the Translation Initiation Site

Having found no evidence of either mRNA primary sequence or secondary structure directing translation of mRNAs without an SD sequence, we finally considered the possibility that lack of secondary structure at or around the start codon promotes faithful translation initiation at the correct AUG [15]. This is conceivable, because 60% of the nucleotides in random RNA sequences and even 60–70% of the nucleotides in natural mRNAs are engaged in base pairing, a value similarly high as for the (highly structured) ribosomal RNAs [16], [17]. Consequently, most of the mRNA sequence is highly structured and not accessible in a single-stranded form [18], [15]. Moreover, in the γ-proteobacterium *Escherichia coli*, it is well established that the stability of mRNA folding downstream of the SD sequence (in the region from −4 to +37) determines translational efficiency [19]. It, therefore, seemed possible that lack of structure around the initiator codon represents the key feature that facilitates SD-independent translation at the correct AUG.

**Figure 2:**
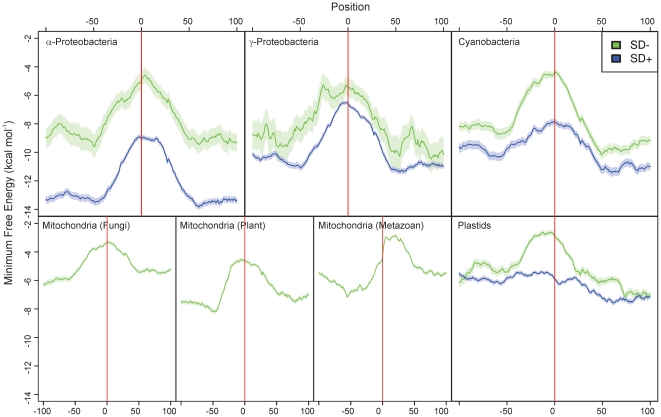
The amount of RNA secondary structure predicted around the start codon in α-proteobacteria, γ-proteobacteria, cyanobacteria, plant, metazoan and fungal mitochondria, and plastids. Position 0 is the first nucleotide of the start codon. Genes without an SD sequence are represented by green curves, those with an SD by blue curves. The line shows the running mean minimum free energy of 5000 genes, the shaded area around it indicates the standard error of the mean. The minimum free energy was determined using a sliding window covering 50 nucleotides. The difference in MFE between the green and blue curves upstream and downstream of the initiation region in the three bacterial groups is largely due to differences in AT-content between genomes within an individual organismal group and a correlation between AT-richness and prevalence of SD-independent translation. The MFE difference disappears when individual genomes are analyzed (Figure S1). Note that, in metazoan mitochondria, the peak of the minimum free energy is shifted into the coding region, because most transcripts in animal mitochondria are leaderless and lack a 5′ UTR.

To test this idea, we randomly selected 5000 genes from each group of prokaryotes known to be capable of SD-independent translation: α-proteobacteria, γ-proteobacteria, cyanobacteria, plastids, metazoan mitochondria, fungal mitochondria and plant mitochondria. The genes were first classified according to the presence or absence of an SD sequence (see Materials and Methods). A running window was passed over a region from 100 nucleotides downstream of the start codon to 100 nucleotides upstream of the start codon and folded to calculate the minimum free energy (MFE; [20]). Interestingly, these analyses revealed that, in all systems where SD-dependent and SD-independent translation co-exist (i.e., all prokaryotes except mitochondria), mRNAs lacking an SD sequence are considerably less structured around the initiator codon than genes possessing an SD sequence (Figure 2 and Figure S1). In α-proteobacteria and γ-proteobacteria, mRNAs with an SD sequence also showed a pronounced maximum of RNA unfoldedness around the translation initiation site (Figure 2). However, this maximum was significantly lower than in mRNAs without an SD sequence (Figure 2 and Figure S1) and, moreover, was much less pronounced in cyanobacterial mRNAs and entirely absent from plastid mRNAs. In contrast, it was similarly high in SD-free mRNAs across all organismal groups (Figure 2). The results were independent of the size of the running window used in these analyses (Figures S2, S3, and S4).

Interestingly, in most organismal groups, the maximum of RNA unfoldedness is centered asymmetrically around the translational start codon, with low folding extending further into the upstream than the downstream region (Figure 2). This correlates well with asymmetric positioning of the initiating 30S ribosomal subunit (covering mRNA positions −35 to +5; [21]). The only exception are metazoan mitochondria, where the MFE peak is shifted into the coding region. This is because most transcripts in animal mitochondria are leaderless and lack a 5′ UTR.

### Experimental Analysis of the Role of mRNA Unfoldedness in SD–Independent Translation

**Figure 3:**
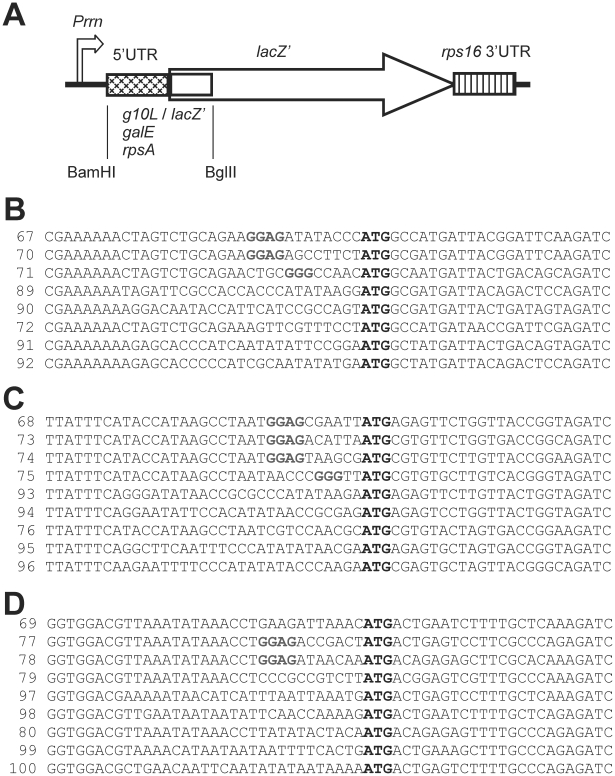
Reporter gene constructs. (A) Schematic map of the *lacZ'* cassette. Promoter (*Prrn*), 3′ UTR (*rps16* 3′ UTR) and coding region (*lacZ'*) are marked. The arrow denotes the direction of transcription. The sequence between the BamHI and BglII restriction sites was exchanged in the various constructs (i. e., the 5′ UTR indicated by the cross-hatched box and the first 21 nt of the coding region indicated by the open box). (B) Sequences from -33 to +26 of the constructs based on the *gene 10* 5′ UTR and the 5′ end of the *lacZ*' coding region. The numbers indicate the pLS plasmid numbers. pLS67 contains the unmutated sequence. (C) Sequences from -33 to +26 of the constructs based on the *galE* 5′ UTR and 5′ end of the coding region. pLS68 harbors the wild-type sequence. (D) Sequences from -33 to +26 of the constructs based on the *rpsA* 5′ UTR and 5′ end of the coding region. pLS69 contains the wild-type sequence. Shine-Dalgarno sequences are shown in bold grey letters, the ATG start codon is indicated by bold black letters.

**Figure 4:**
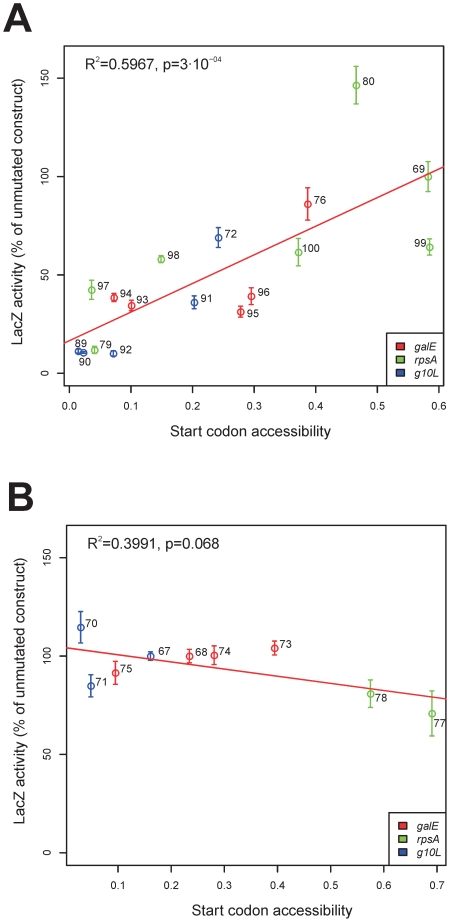
Experimental testing of the unfoldedness hypothesis for SD–independent translation. (A) Correlation between LacZ activity and accessibility of the start codon in constructs without a SD sequence. (B) Lack of a significant correlation between LacZ activity and accessibility of the start codon in constructs with a SD sequence. The 5′ UTRs on which the individual constructs were based are indicated by color-coding. The LacZ activity was normalized to the activity of the unmutated construct (*g10L*: pLS67; *galE*: pLS68; *rpsA*: pLS69; cp. Figure 3). The start codon accessibility was calculated as the probability that every position of the start codon is unbound and was determined by RNAplfold [34] using a window size of 50 nt and an unbound region size of 3 nt.

Having obtained strong bioinformatics support of RNA unfoldedness being the major determinant of start codon recognition in SD-independent translation initiation, we next wanted to provide direct experimental confirmation. To this end, we constructed a large series of reporter gene fusions based on the bacterial *lacZ'* gene (encoding β-galactosidase), in which we (i) mutationally manipulated start codon accessibility by changing RNA structure and (ii) created or eliminated an SD motif (Figure 3). Three natural 5′ UTR sequences and 5′ coding sequences were used: (i) the 5′ UTR from *gene 10* of phage T7 (*gene 10* leader, *g10L*) combined with the 5′ coding sequence from *lacZ*', (ii) the 5′ UTR and 5′ coding sequence from the *Escherichia coli galE* gene, and (iii) the 5′ UTR and 5′ coding sequence from the *E. coli rpsA* gene (Figure 3). While the *g10L* and *galE* 5′ UTRs contain canonical SD sequences, the *rpsA* mRNA does not contain an SD motif and has been demonstrate to be translated in an SD-independent manner [6]. For all three sequences, we computationally designed a series of mutant versions that either possessed or lacked an SD motif (Figure 3) and, moreover, varied in their degree of RNA foldedness and start codon accessibility over a wide range (Figure 4, Figures S5 and S7). All constructs were introduced into *Escherichia coli* cells and LacZ protein accumulation was measured by an enzyme activity assay using o-nitrophenyl-β-d-galactopyranoside (o-NPG) as synthetic substrate. When the LacZ protein accumulation from all constructs lacking a SD sequence was plotted against the degree of RNA foldedness around the translational start codon, a strong positive correlation between LacZ accumulation and start codon accessibility was observed (Figure 4A). This correlation was statistically highly significant with a p-value of 3.0 · 10^−04^ and a coefficient of correlation (R^2^) of 0.6. In contrast, no such correlation was seen in the constructs containing the SD motif (Figure 4B). Also, there was no correlation between the accessibility of the SD and the LacZ activity (Figure S6). Most importantly, mutational elimination of the SD sequence from the *g10L* and *galE* 5′ UTRs (as, e.g., in constructs 89 and 93) drastically reduced LacZ expression and this effect could be compensated for by introduction of additional mutations that reduce RNA structure at the start codon (with LacZ expression in the least folded construct 76 reaching 86.1% of wild-type levels). Conversely, mutations that increase RNA structure at the start codon in the *rpsA* mRNA (that naturally lacks an SD motif) strongly reduced LacZ accumulation to as low as 11.9% of wild-type levels in the highly folded construct 79; Figure 3 and Figure 4). Also, the minimum free energy in the region surrounding the translational start codon was negatively correlated with LacZ protein accumulation in the constructs lacking an SD sequence, but no such correlation was observed for the constructs with an SD sequence (Figure S7).

Potential correlations with a number of other properties of the mRNAs were explored, including AU content (Figure S8), codon adaptation index (Figure S9) and tRNA abundance (Figure S10). None of these parameters were significantly correlated with LacZ protein accumulation, suggesting that they do not appreciably influence translation rates. The extent of base-pairing between the 5′ UTR and the ASD in the 3′ end of the 16S rRNA was positively correlated with LacZ expression (Figure S11). This was expected because of the high number of constructs that have an SD sequence (and are largely insensitive to RNA structure) and the high number of constructs that lack an SD sequence (and, consistent with the unfoldedness hypothesis for SD-independent translation, are sensitive to RNA structure; Figure 4).

### Comparison of RNA Structuredness around Initiator AUG Codons and Internal AUG Codons

**Figure 5:**
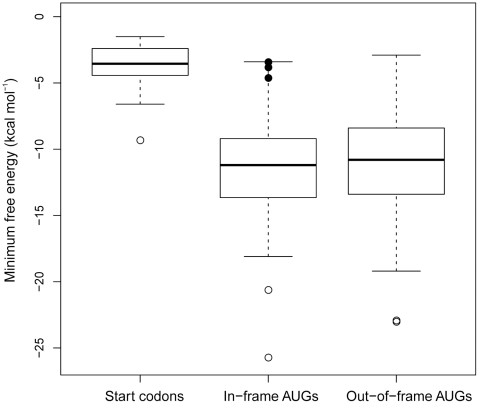
Comparison of the minimum free energy (MFE) values of start codons, in-frame AUG codons, and out-of-frame AUG triplets in the *Escherichia coli* genome. The middle line in the box is the median, the next two are the 1^st^ and 3^rd^ quartiles, the whiskers extend to 1.5 times the interquartile range of the box. Outliers are indicated by open circles. The outlier in the start codon group is the MFE value for the start codon of *trmD* (see text for details). Three in-frame AUGs (filled black circles) of *murC*, *metG* and *trmD*, respectively, were singled out for their close proximity and MFE to the start codon. Based on the low amount of structure around them, these AUGs are candidates for alternative translation initiation sites.

An immediate prediction from the unfoldedness hypothesis for SD-independent translation is that internal AUG triplets should be much less accessible to the ribosome than AUG start codons. To test whether lack of structure is the distinguishing feature that sets apart initiator AUG codons from internal AUG triplets and, in this way facilitates start codon recognition with high selectivity, we analyzed all genes in the *E. coli* gene set that lack an SD sequence and calculated the MFE in a 50 nt window surrounding all AUG triplets found in these sequences. Strikingly, the amount of structure around internal AUG triplets was found to be much higher than that around initiator AUGs (Figure 5). A single outlier was the annotated start codon of the *trmD* mRNA encoding the tRNA-modifying enzyme tRNA m(1)G37 methyltransferase (Figure 5). *trmD* is part of an operon containing two highly expressed ribosomal protein genes (*rpsP* and *rplS* encoding ribosomal proteins S16 and L19, respectively). Although being part of the same tetracistronic transcript, expression of *trmD* at the protein level was found to be 40-fold lower than expression of *rpsP* and *rplS* [22], suggesting strong translation control. It, therefore, seems conceivable that the exceptionally strong secondary structure around the start codon in *trmD* has evolved to keep protein production low in spite of the large amounts of transcripts needed for the massive synthesis of the ribosomal proteins S16 and L19 [22]. Interestingly, we also discovered three cases, in which in-frame AUG codons believed to be internal are located very close to the annotated start codon (filled circles in Figure 5) and, unlike other internal AUG triplets, display a relatively low amount of structure around them. We, therefore, propose that these AUGs are good candidates for alternative translation initiation sites or may even be the only real start codon. Remarkably, one of these lowly structured AUGs is an in-frame AUG codon in the *trmD* mRNA, just 10 codons downstream of the annotated start codon.

## Discussion

In this work, we have addressed the question how specific start codon recognition and efficient translation initiation can occur in the absence of a ribosome-binding site. Our genome-wide searches for Shine-Dalgarno-independent translation in bacterial and organellar genomes revealed that a large fraction of transcripts is translated in a Shine-Dalgarno-independent manner in all prokaryotic systems (Figure 1). The extent of Shine-Dalgarno-independent translation is variable between different groups of organisms, ranging from approximately 15% of the genes in the genome of γ-proteobacteria to 100% of the genes in mitochondrial genomes.

Our data provide strong bioinformatics as well as experimental support for RNA unfoldedness being the major requirement for efficient start codon recognition in SD-independent translation initiation. At first glance, it may seem inconceivable that single-strandedness is sufficient to define an AUG triplet as initiator codon. However, it is important to realize that most of the coding sequence of mRNAs in both prokaryotes [18], [15] and eukaryotes [23] is highly structured and, therefore, not accessible in a single-stranded form. Therefore, presence of an AUG codon in an unstructed region can unambiguously define the correct translation initiation site (Figure 5). Our genome-wide analyses indicate that, at least in some prokaryotic systems, there is also a selective pressure towards start codon accessibility in SD-dependent translation (Figure 2 and Figure S1). This is consistent with the idea that the stability of mRNA folding near the SD sequence can influence translational efficiency [19] and may facilitate facile switching between SD-dependent and SD-independent translation in evolution. However, in all systems, the selective pressure towards start codon accessibility is considerably lower in mRNAs with an SD sequence than in mRNAs without an SD sequence. Moreover, it appears to be very low in cyanobacteria and entirely absent from plastids (Figure 2). Strikingly, cyanobacteria and plastids are the two systems with the by far highest prevalence of SD-independent translation (Figure 1). This suggests contrasting modes of genome-wide selection for start codon accessibility in SD-dependent translation initiation. However, for SD-independent translation, start codon accessibility appears to be a general requirement in all prokaryotic systems. It will be interesting to identify the evolutionary forces underlying these genome-wide differences in the utilization of SD-independent translation and its possible co-evolution with structural constraints in SD-dependent translation.

The mechanism how the ribosome recognize the start codon in the absence of an SD sequence could be conceptually simple. The initial binding of the ribosome to the mRNA is, to a large degree, sequence independent [24]. In SD-dependent translation, the SD sequence likely mediates the subsequently occurring correct positioning of the ribosome on the mRNA. In SD-independent translation, this positioning function may be fulfilled by a single-stranded RNA region around the initiation codon. Also, single-strandedness is likely to facilitate recognition of the AUG start codon by the anticodon of the initiator tRNA-fMet.

The low amount of structure in the 5′ part of the coding region of leaderless mRNAs in metazoan mitochondria ([25]; Figure 2) could indicate that SD-independent translation initiation is mechanistically similar to the translation of at least some leaderless mRNAs. Translation initiation on leaderless mRNAs in animal mitochondria has been found to be independent of the presence of the large subunit of the ribosome [26]. A somewhat different mechanism may operate in kasugamycin-treated bacterial cells, where a unique type of reduced ribosomes (61S ribosomes lacking several proteins of the small ribosomal subunit) has been shown to preferentially translate leaderless mRNAs [27].

In summary, our findings provide a plausible mechanism for start codon recognition in SD-independent translation. In addition, they should prove useful in predicting translational efficiency on a genome-wide scale and in aiding the design and optimization of transgene expression constructs in diverse groups of prokaryotes.

## Materials and Methods

### Sequence Motif Discovery

To search for presence of the Shine-Dalgarno sequence motif, all available bacterial and plastid sequences in RefSeq release 42 were downloaded (ftp.ncbi.nih.gov/refseq/release/; [28]). To filter out potential pseudogenes and open reading frames of unclear functional significance, only genes that were present in at least 50% of the members within each class of genomes (bacterial and plastid) were considered, resulting in 160,340 bacterial sequences and 10,420 plastid sequences. For each class, 1000 genes were randomly selected and MEME [12] was used to identify conserved motifs in the 5′ UTR from positions -22 to -2 nucleotides upstream of the start codon. In bacteria, the most significant motif discovered was the SD sequence in 608 of the sequences. All other discovered motifs occurred in less than 20 of the bacterial sequences. In plastids, the most significant motif discovered was also the SD sequence and occurred in 281 of the plastid sequences. The remaining motifs were due to identical or highly similar 5′ UTR regions of homologous genes. The position-specific scoring matrices (PSSM) for the SD sequences were used with MAST [29] to further search for the SD motif in the remaining genes resulting in 88,714 (55.3%) bacterial and 3958 (38.0%) plastid genes that matched the PSSM with a p-value of less than 0.01. To search for alternative motifs to the SD sequence, we removed all sequences whose 5′ UTR region matched the Shine-Dalgarno PSSMs (resulting in 71,626 bacterial and 6462 plastid sequences without Shine-Dalgarno sequences) and repeated the original procedure.

In analogous searches, the Amadeus motif discovery platform [13] was used to identify conserved motifs in the 5′UTR from positions −22 to −2 nucleotides upstream of the start codon. For both classes of genomes, a random background was generated by shuffling the sequences 5 times resulting in 5000 shuffled sequences and conserving the nucleotide composition. The 5′ UTRs containing SD sequences were identified using MAST [29] and removed and the remaining sequences were re-analyzed using Amadeus. The background in the second round was generated in the same manner as in the first. The 5′ UTRs containing SD sequences were used as the genomic background. The p-values were corrected for multiple testing through the permutation method using 20 permutations of the input sequences.

### Structural Motif Discovery

Structural motifs were sought for in the same bacterial and plastid datasets as the sequence motifs. From the sequences belonging to each class, 1000 random sequences were chosen. Each of these sequences were folded from −25 nucleotides upstream to 25 nucleotides downstream of the start codon using RNAshapes [14] to obtain an abstract RNA structure that disregards stem length and loop size. All possible structural motifs were calculated from the resulting structures. This procedure was repeated a further 1000 times, randomly shuffling the non-coding region whilst preserving the dinucleotide frequency with each iteration. For each structural motif calculated from the original sequences, a p-value was calculated from the number of observed occurrences of the motif versus the number of expected occurrences given sequences with the same dinucleotide composition and coding sequence.

### 16S rRNA tail/5′ UTR Hybridization

Of all available genes in RefSeq release 42, only those that were annotated in at least 50% of the available genomes were used. The sequences were trimmed at −125 and overlapping upstream coding regions were removed. Sequences containing ambiguous nucleotides were excluded from the analysis. This resulted in 14,659 α-proteobacterial, 31,496 γ-proteobacterial, 6202 cyanobacterial and 11,238 plastid genes. The ASD sequence (CCUCCU) was computationally hybridized to the 5′ UTR from −22 to −2 nucleotides upstream of the start codon using the free2bind RNA-RNA hybridization algorithm [30]. A relaxed threshold of 0 kcal mol^−1^ for calling Shine-Dalgarno sequences was applied.

### Analysis of RNA Structure around the Start Codon

For an *in silico* analysis of RNA secondary structure formation at and/or near the translation initiation codon, we used genes from the RefSeq database that were present in at least 50% of the bacterial, plastid, metazoan mitochondrial, fungal mitochondrial and plant mitochondrial genomes. For each class of genomes, 5000 genes were randomly selected, with each member being chosen with equal probability, for further analysis. The hybridization energy of the anti-Shine-Dalgarno sequence in the 16S rRNA in each of the genomes to the selected genes was then calculated using the annotated 16S rRNA. If the 16S rRNA was not or incorrectly annotated, the *Escherichia coli* sequence was used. As there is no consensus, we applied strict conditions for the presence and absence of an SD sequence. The presence was defined as a hybridization energy of less than −4.4 kcal mol^−1^ and the absence as greater than 0 kcal mol^−1^. A running window of 50 nucleotides was then passed over a region from 100 nucleotides downstream of the start codon to 100 nucleotides upstream of the start codon and folded using RNAfold [20] to calculate the minimum free energy (MFE). The sequences were separated into SD positive and SD negative groups and the mean and standard error of the mean were calculated.

To compare initiator AUG codons with internal AUG triplets in *E. coli* genes lacking an SD sequence (determined using free2bind), all *E. coli* K12 (AC_000091) genes from −125 from the start codon to +125 from the stop codon were extracted from GenBank applying the same criteria as described under “Sequence motif discovery”. Any non-coding regions (both upstream and downstream) that overlapped with other coding regions from the neighboring genes were removed. The hybridization energy of the ASD was calculated (see 16S rRNA tail/5′ UTR hybridization) for the remaining genes with an intact −22 to −2 region upstream of the annotated start codon. For all genes predicted to contain no SD sequence, the minimum free energy was calculated in a 50 nt window surrounding all AUGs found in the sequence. The AUGs were then split into annotated start codons, in-frame AUG codons and out-of-frame AUG triplets.

### Cloning Procedures and Computational Design of Constructs with Altered RNA Structures

All constructs are based on the previously published vector pBSU0 [31]. A BglII restriction site was inserted between position +21 and +22 of the coding region of *lacZ*' by amplifying the gene using primers PLS67for and PLS67rev (Table S1). The amplification product was digested with NcoI and XbaI (the corresponding restriction sites are present at the start codon and after the stop codon of *lacZ'*, respectively) and inserted into the similarly cut pBSU0. The resulting plasmid pLS67 contains the *Prrn* promoter from *Nicotiana tabacum*, the *gene 10* 5′ UTR from phage T7, the coding region of *lacZ*' from *Escherichia coli*, and the *rps16* 3′ UTR from *Nicotiana tabacum* [31]. A sequence comprising the *galE* 5′ UTR (from −35 to −1, according to [6]) and the first 21 nt of the *galE* coding region from *Escherichia coli* was produced by annealing two overlapping oligonucleotides (PLS68for and PLS68rev; Table S1) followed by amplification of the double-stranded sequence with Phusion DNA polymerase (Finnzymes, Hess. Oldendorf, Germany). The PCR product was digested with BamHI and BglII and inserted into vector pLS67 digested with the same enzymes, generating plasmid pLS68. Using a similar strategy, plasmid pLS69 was constructed based on the sequence comprising the 5′ UTR (−91 to −1; [6]) and the first 21 nt of the *rpsA* coding region from *Escherichia coli* (using primers PLS69for and PLS69rev; Table S1). The *galE* and *rpsA* sequences were taken from the genome sequence of *Escherichia coli* strain K-12 substrain W3110 ([32]; database accession number AC_000091).

The sequence of the 5′ UTR and the first 21 nt of the coding regions in constructs pLS70 to pLS72 and pLS89 to pLS92 was based on the *gene 10* 5′ UTR and the 5′ end of the *lacZ*' coding region. In constructs pLS73 to pLS76 and pLS93 to pLS96, the sequence is derived from the *galE* 5′ UTR and 5′ end of the coding region and in constructs pLS77 to pLS80 and pLS97 to pLS100, on the *rpsA* 5′ UTR and 5′ end of the coding region. In pLS77 and pLS78, an SD sequence was created by an A to G mutation at position −10.

To alter the RNA structure, the sequence between the SD sequence and nucleotide +21 in constructs pLS70, pLS73, pLS74, pLS77 and pLS78 was mutated. In pLS71 and pLS72, the mutated sequence ranges from nucleotides −12 to +21, in pLS75 and pLS76 from −10 to +21, in pLS79 and pLS80 from −11 to +21, in pLS89 to pLS100 from −25 to +21. The sequences with modified RNA structure were generated using a stochastic search strategy similar to simulated annealing. The score used to optimize sequences was calculated using the difference between the actual and target minimum free energies around the start codon and the actual and target hybridization energies of the ASD sequence in the *Escherichia coli* 16S rRNA 3′ tail to nucleotides −22 to −2 upstream of the start codon. In each iteration of the algorithm, a random position of the manipulable region in the sequence was chosen for mutation. We used two different approaches when the random position was in the non-coding region. For constructs pLS70 to pLS80, the position was randomly mutated into a new nucleotide. For constructs pLS89 to pLS100, the non-coding region was shuffled whilst preserving the dicodon frequency. If the random position was in the coding region, the affected codon was replaced with a synonymous codon using a probability derived from the *E. coli* codon usage table. For each construct, 100 optimized sequences were generated and the top ten were further manually examined for suitability in experimentation based on the predicted structure and sequence composition. pLS72, pLS74, pLS76, pLS78, pLS80, pLS91, pLS92, pLS95, pLS96, pLS99 and pLS100 were designed to have a low amount of structure around the start codon, whereas pLS70, pLS71, pLS73, pLS75, pLS77, pLS79, pLS89, pLS90, pLS93, pLS94, pLS97 and pLS98 were designed to have a high amount of structure. The mutated sequences to generate plasmids pLS70 to pLS80 and pLS89 to pLS100 were inserted into pLS67 using the same strategy as for pLS68. Primer numbers in Table S1 correspond to construct numbers. For plasmids pLS77 to pLS80 and pLS97 to pLS100, primer PLS69rev was used as reverse primer.

### o-NPG Assay and Data Evaluation

All constructs were transformed into *Escherichia coli* strain SURE2 (Stratagene/Agilent Technologies, Waldbronn, Germany). o-NPG (o-nitrophenyl-β-d-galactopyranoside; Sigma-Aldrich, Munich, Germany) assays was performed as described previously [33].

The constructs were separated into constructs with and without SD sequences based on the hybridization energy between the ASD sequence in the *Escherichia coli* 16S rRNA 3′ tail and the region in the mRNA 5′ UTR from nucleotides −22 to −2 upstream of the start codon. The threshold for presence of an SD interaction was set to 0 kcal mol^−1^. The accessibility of the start codon in each construct was calculated as the probability that the start codon was unbound and was determined using RNAplfold [34]. The Codon Adaptation Index (CAI) was calculated from the relative synonymous codon usage (RSCU) table for *Escherichia coli*. The RSCU is the observed frequency of a codon divided by frequency expected under assumption of equal usage of synonymous codons for an amino acid. The CAI has been used to predict the expression level of a gene and assesses the extent to which selection has been successful at moulding the pattern of codon usage [35]. The correlation between tRNA abundance and LacZ activity was assessed using published tRNA abundance measurements [36] and calculating the expected expression for each construct. The average minimum free energy (MFE) of the manipulated region, from −25 nucleotides upstream to +21 nucleotides downstream of the start codon, was calculated for each construct using RNAfold [20].

## Supporting Information

Figure S1 Amount of RNA secondary structure predicted around the start codons in the genomes of (A) the α-proteobacterium *Caulobacter crescentus* (NC_002696.2; 17.4% of the genes lacking an SD) and (B) the γ-proteobacterium *Escherichia coli* (AC_000091; 10.7% of the genes lacking an SD). Position 0 is the first nucleotide of the start codon. Genes without an SD sequence are represented by green curves, those with an SD by blue curves. The line shows the running mean minimum free energy, the shaded area around it indicates the standard error of the mean. The minimum free energy was determined using a sliding window covering 50 nucleotides.
(TIF)

Figure S2 The amount of RNA secondary structure predicted around the start codon in α-proteobacteria (A, C, E) and γ-proteobacteria (B, D, F). Position 0 is the first nucleotide of the start codon. Genes without an SD sequence are represented by green curves, those with an SD by blue curves. The minimum free energy was determined using a sliding window covering 50 (A, B), 75 (C, D), and 100 (E, F) nucleotides. The predicted reduced amount of structure around the start codon is independent of the size of the window.
(TIF)

Figure S3 The amount of RNA secondary structure predicted around the start codon in cyanobacteria (A, C, E) and plastids (B, D, F). Position 0 is the first nucleotide of the start codon. Genes without an SD sequence are represented by green curves, those with an SD by blue curves. The minimum free energy was determined using a sliding window covering 50 (A, B), 75 (C, D), and 100 (E, F) nucleotides. The predicted reduced amount of structure around the start codon is independent of the size of the window.
(TIF)

Figure S4 The amount of RNA secondary structure predicted around the start codon in mitochondria of fungi (A, D, G), plants (B, E, H), and metazoa (C, F, I). Position 0 is the first nucleotide of the start codon. The minimum free energy was determined using a sliding window covering 50 (A–C), 75 (D–F), and 100 (G–I) nucleotides. The predicted reduced amount of structure around the start codon is independent of the size of the window.
(TIF)

Figure S5 Sequences (from −100 to +100) and secondary structures with the minimum free energy for all constructs. Start codon and Shine-Dalgarno sequence are shown in bold. An opening bracket indicates that base pairing occurs between the represented region and the region of the matching closing bracket, a point indicates a stretch of unpaired nucleotides. The calculated stability of each structure is indicated by the free energy value behind the construct name. Note that the structures shown here do not accurately capture the accessibility of the start codon, because (i) the most stable fold can depend on the length of the input sequence and (ii) RNA molecules are structurally dynamic and constantly shift between alternative conformations.
(TIF)

Figure S6 Correlation of LacZ activity and RNA structure in constructs with SD sequence. (A) Correlation between the accessibility of the Shine-Dalgarno sequence and LacZ activity. (B) Correlation between the minimum free energy of the region comprising 50 nt around the start codon and LacZ activity.
(TIF)

Figure S7 Correlation between the minimum free energy of the region comprising 50 nt around the start codon and LacZ activity. (A) Correlation in constructs without SD. (B) Correlation in constructs with SD.
(TIF)

Figure S8 Correlation between AU content in the −30 to −5 region and LacZ activity in reporter gene constructs. Lack of a significant correlation excludes the possibility that the efficiency of translation initiation depends on binding of the ribosomal protein S1 via AU-rich sequences in the 5′ UTR [9], [11]. (A) Correlation between AU content and LacZ activity in constructs without SD sequence. (B) Correlation between AU content and LacZ activity in constructs with SD sequence.
(TIF)

Figure S9 Correlation between codon adaptation index (CAI) and LacZ activity in reporter gene constructs without SD sequence. The Codon Adaptation Index (CAI) was calculated from the relative synonymous codon usage (RSCU) table for *Escherichia coli*. (A) Correlation between CAI and LacZ activity in constructs without SD. (B) Correlation between CAI and LacZ activity in constructs with SD sequence.
(TIF)

Figure S10 Correlation between tRNA abundance and LacZ activity in reporter gene constructs without SD sequence. Published tRNA abundance measurements [36] were used to calculate the expected expression for each construct. (A) Correlation between tRNA abundance and LacZ activity in constructs without SD. (B) Correlation between tRNA abundance and LacZ activity in constructs with SD sequence.
(TIF)

Figure S11 Correlation between LacZ activity and strength of Shine-Dalgarno-type binding between the 5′ UTR (from position -22 to -2) and the ASD (CCUCCU) in the 16S rRNA tail (hybridization energies given in kcal mol^−1^). There is a significant correlation, because there are many constructs without SD, but also an inaccessible start codon (i. e., constructs that were designed to have low translational activity).
(TIF)

Table S1 List of oligonucleotides used in this study. Primer numbers correspond to construct numbers.
(DOC)
