# Supplementary figures and images for: Local Absence of Secondary Structure Permits Translation of mRNAs that Lack Ribosome-Binding Sites
Source: PLoS Genet. 2011 Jun 23;7(6):e1002155. doi: 10.1371/journal.pgen.1002155 (PMC3121790; doi:10.1371/journal.pgen.1002155)

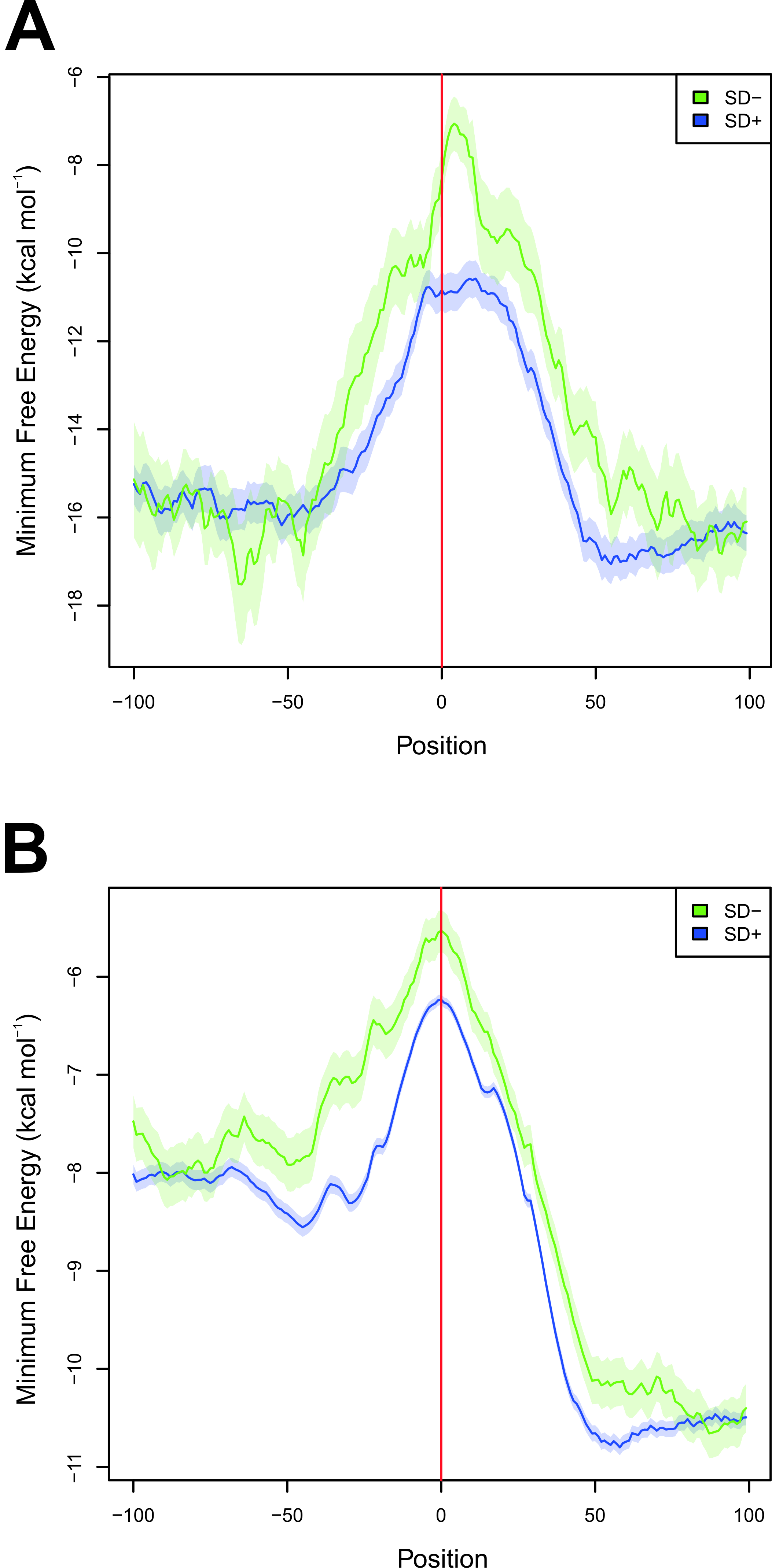

Supplement: Figure S1 — Amount of RNA secondary structure predicted around the start codons in the genomes of (A) the α-proteobacterium Caulobacter crescentus (NC_002696.2; 17.4% of the genes lacking an SD) and (B) the γ-proteobacterium Escherichia coli (AC_000091; 10.7% of the genes lacking an SD). Position 0 is the first nucleotide of the start codon. Genes without an SD sequence are represented by green curves, those with an SD by blue curves. The line shows the running mean minimum free energy, the shaded area around it indicates the standard error of the mean. The minimum free energy was determined using a sliding window covering 50 nucleotides. (TIF) [file pgen.1002155.s001.tif]

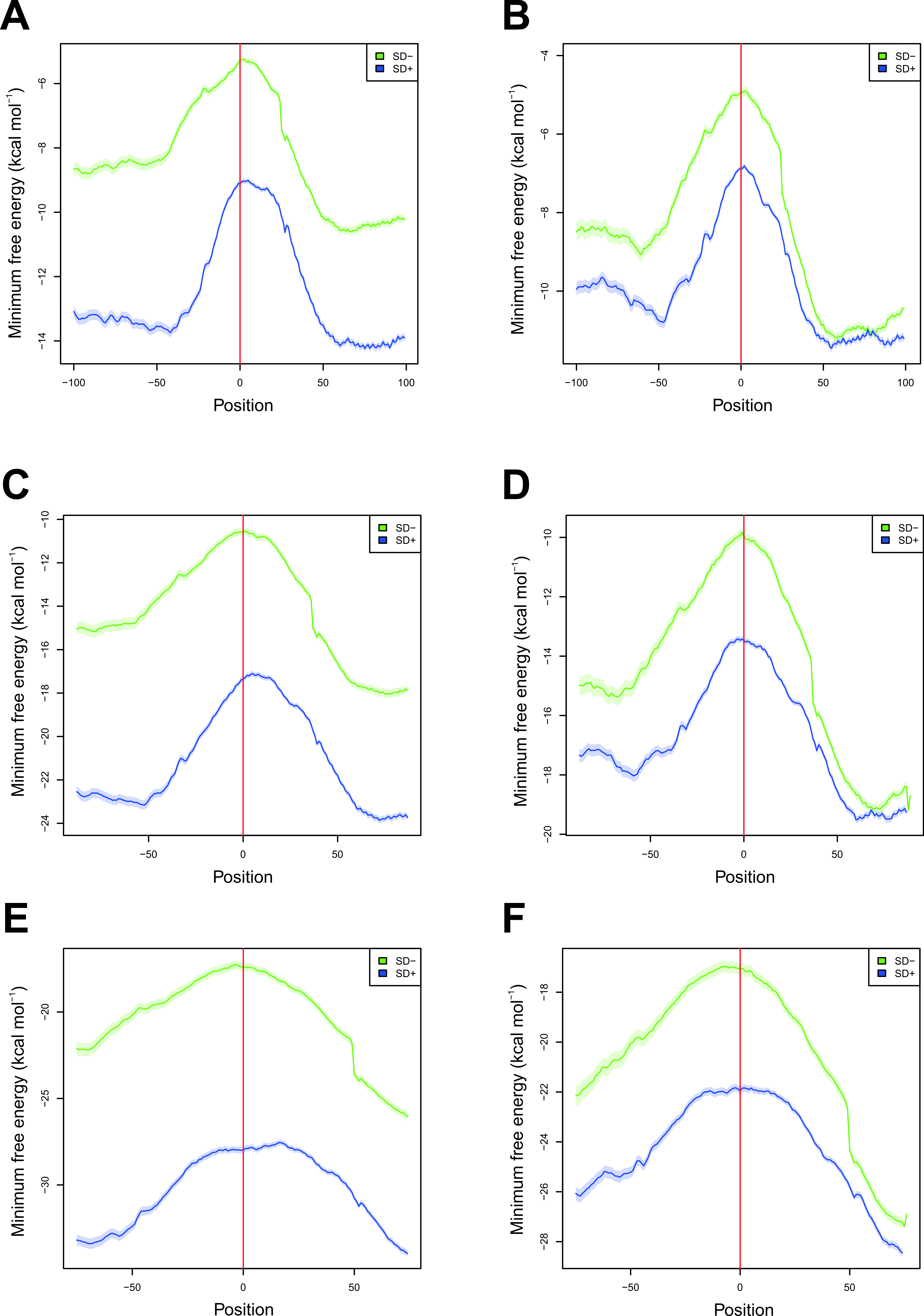

Supplement: Figure S2 — The amount of RNA secondary structure predicted around the start codon in α-proteobacteria (A, C, E) and γ-proteobacteria (B, D, F). Position 0 is the first nucleotide of the start codon. Genes without an SD sequence are represented by green curves, those with an SD by blue curves. The minimum free energy was determined using a sliding window covering 50 (A, B), 75 (C, D), and 100 (E, F) nucleotides. The predicted reduced amount of structure around the start codon is independent of the size of the window. (TIF) [file pgen.1002155.s002.tif]

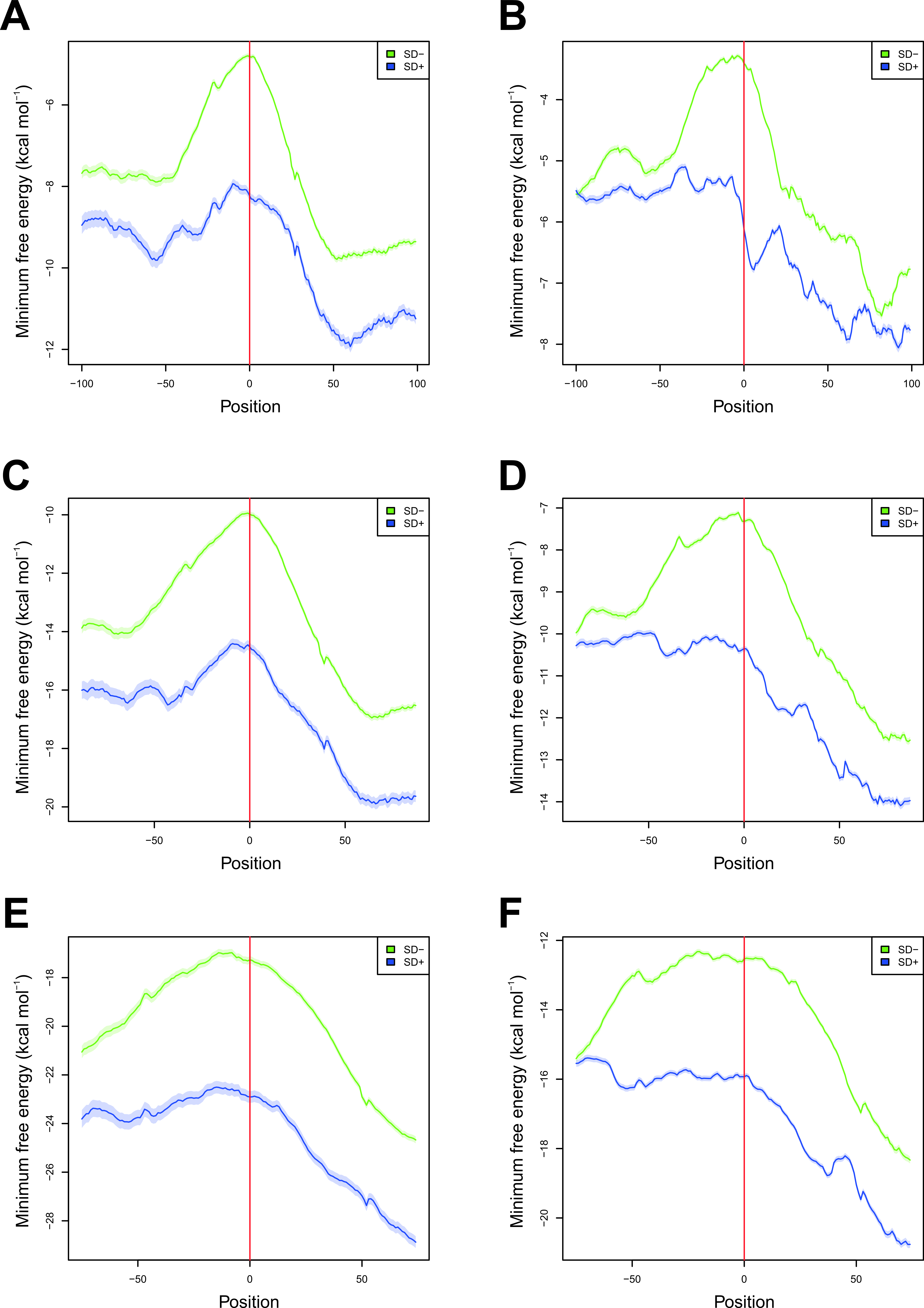

Supplement: Figure S3 — The amount of RNA secondary structure predicted around the start codon in cyanobacteria (A, C, E) and plastids (B, D, F). Position 0 is the first nucleotide of the start codon. Genes without an SD sequence are represented by green curves, those with an SD by blue curves. The minimum free energy was determined using a sliding window covering 50 (A, B), 75 (C, D), and 100 (E, F) nucleotides. The predicted reduced amount of structure around the start codon is independent of the size of the window. (TIF) [file pgen.1002155.s003.tif]

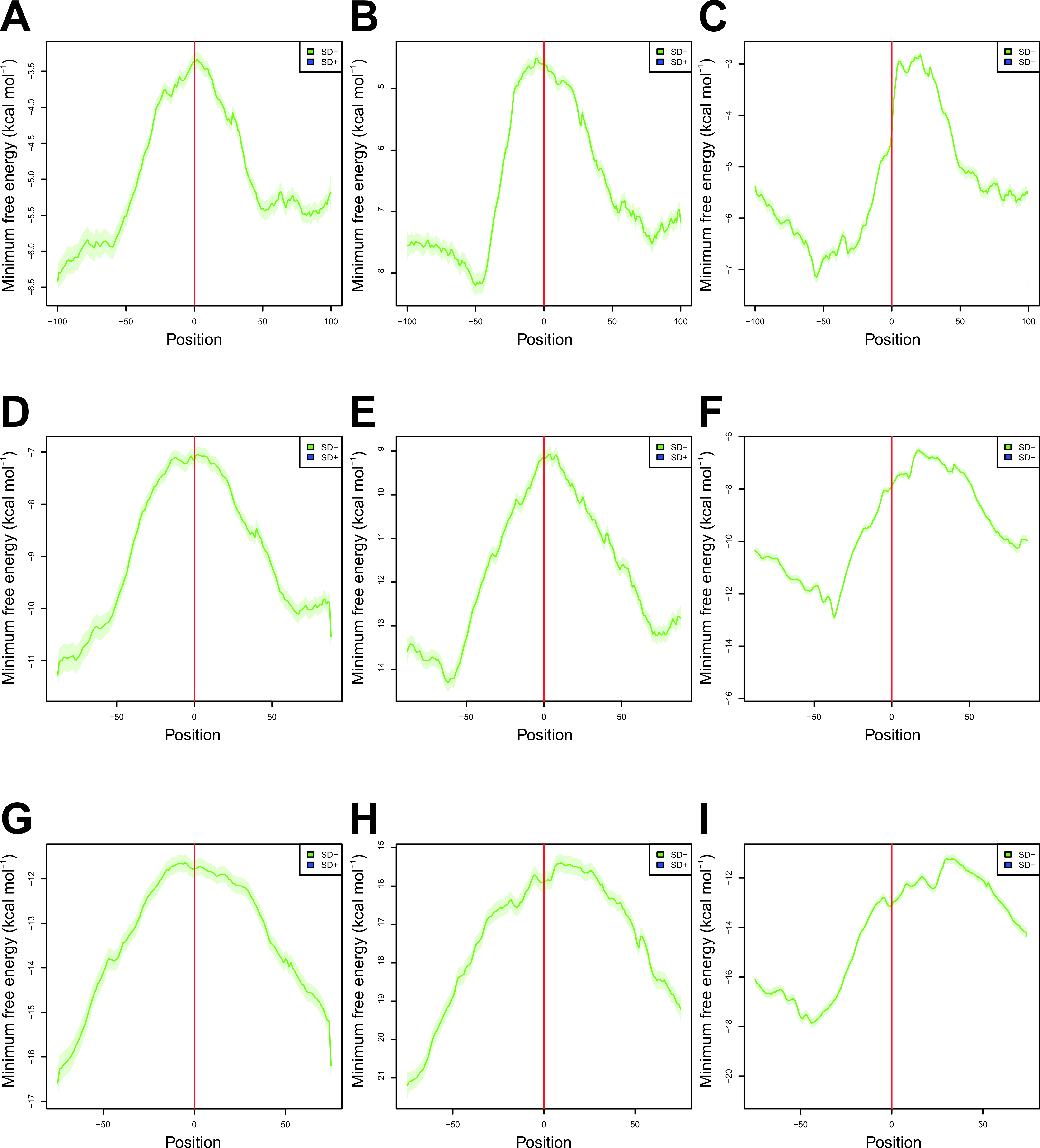

Supplement: Figure S4 — The amount of RNA secondary structure predicted around the start codon in mitochondria of fungi (A, D, G), plants (B, E, H), and metazoa (C, F, I). Position 0 is the first nucleotide of the start codon. The minimum free energy was determined using a sliding window covering 50 (A–C), 75 (D–F), and 100 (G–I) nucleotides. The predicted reduced amount of structure around the start codon is independent of the size of the window. (TIF) [file pgen.1002155.s004.tif]

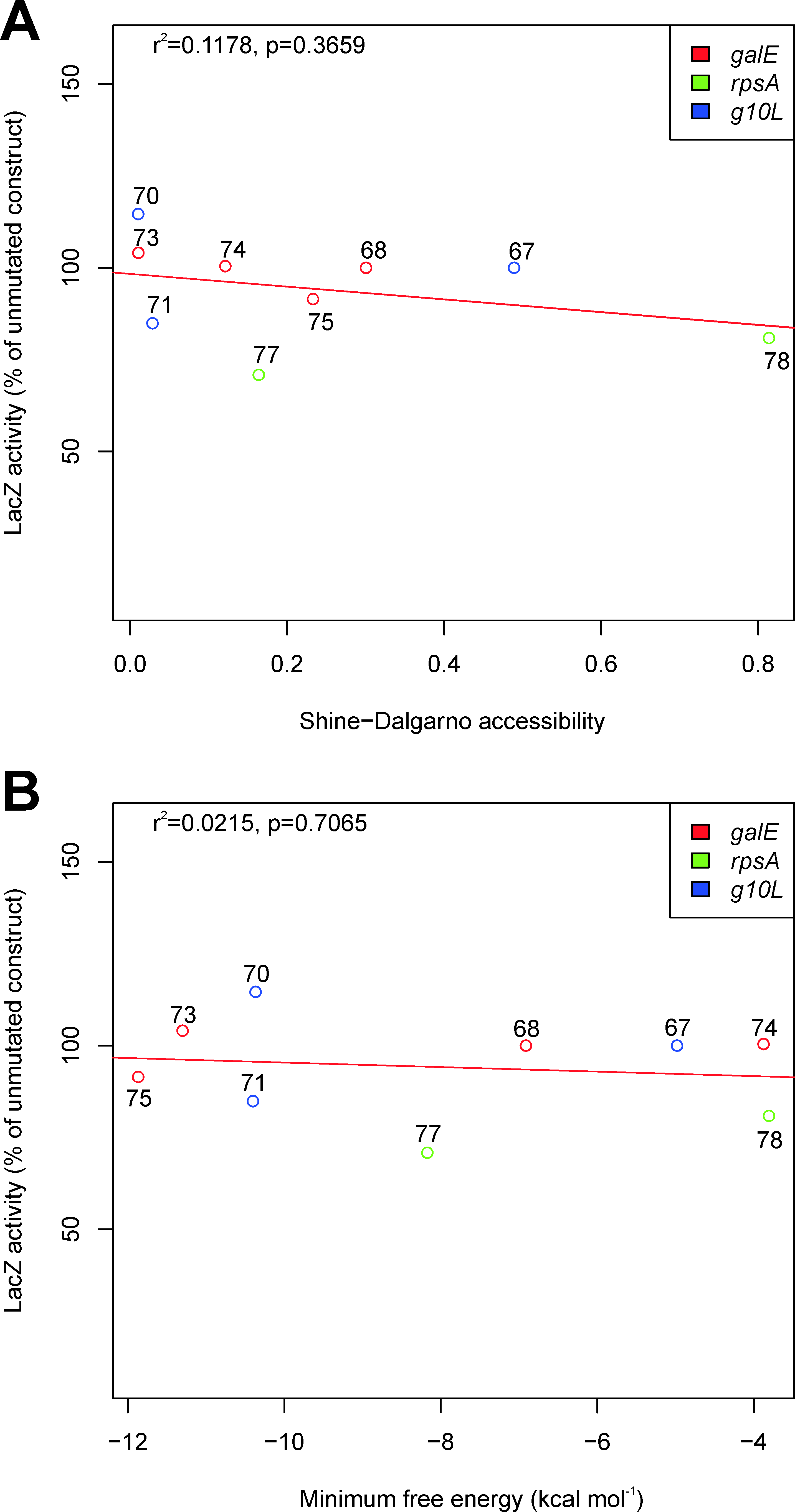

Supplement: Figure S6 — Correlation of LacZ activity and RNA structure in constructs with SD sequence. (A) Correlation between the accessibility of the Shine-Dalgarno sequence and LacZ activity. (B) Correlation between the minimum free energy of the region comprising 50 nt around the start codon and LacZ activity. (TIF) [file pgen.1002155.s006.tif]

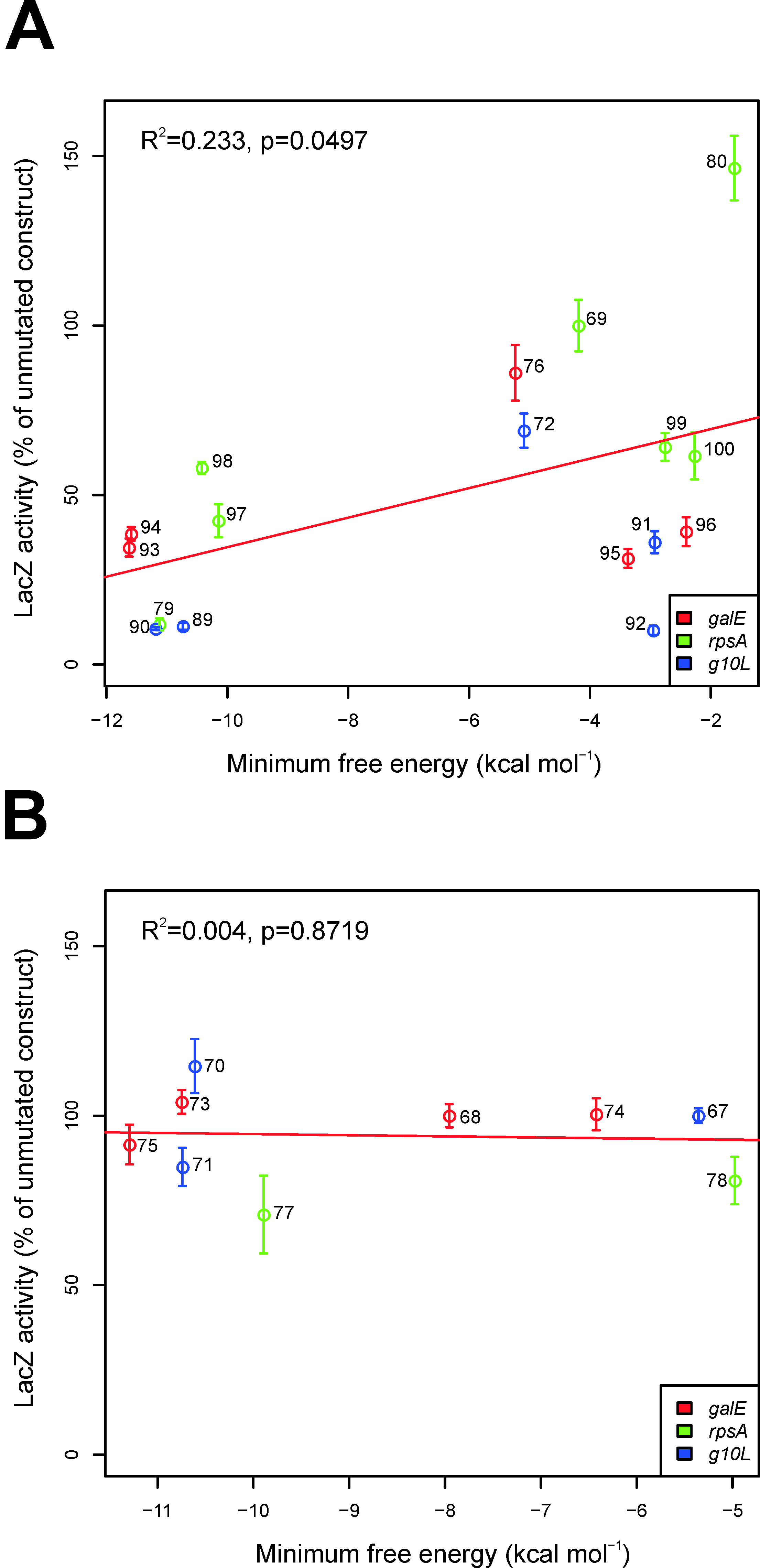

Supplement: Figure S7 — Correlation between the minimum free energy of the region comprising 50 nt around the start codon and LacZ activity. (A) Correlation in constructs without SD. (B) Correlation in constructs with SD. (TIF) [file pgen.1002155.s007.tif]

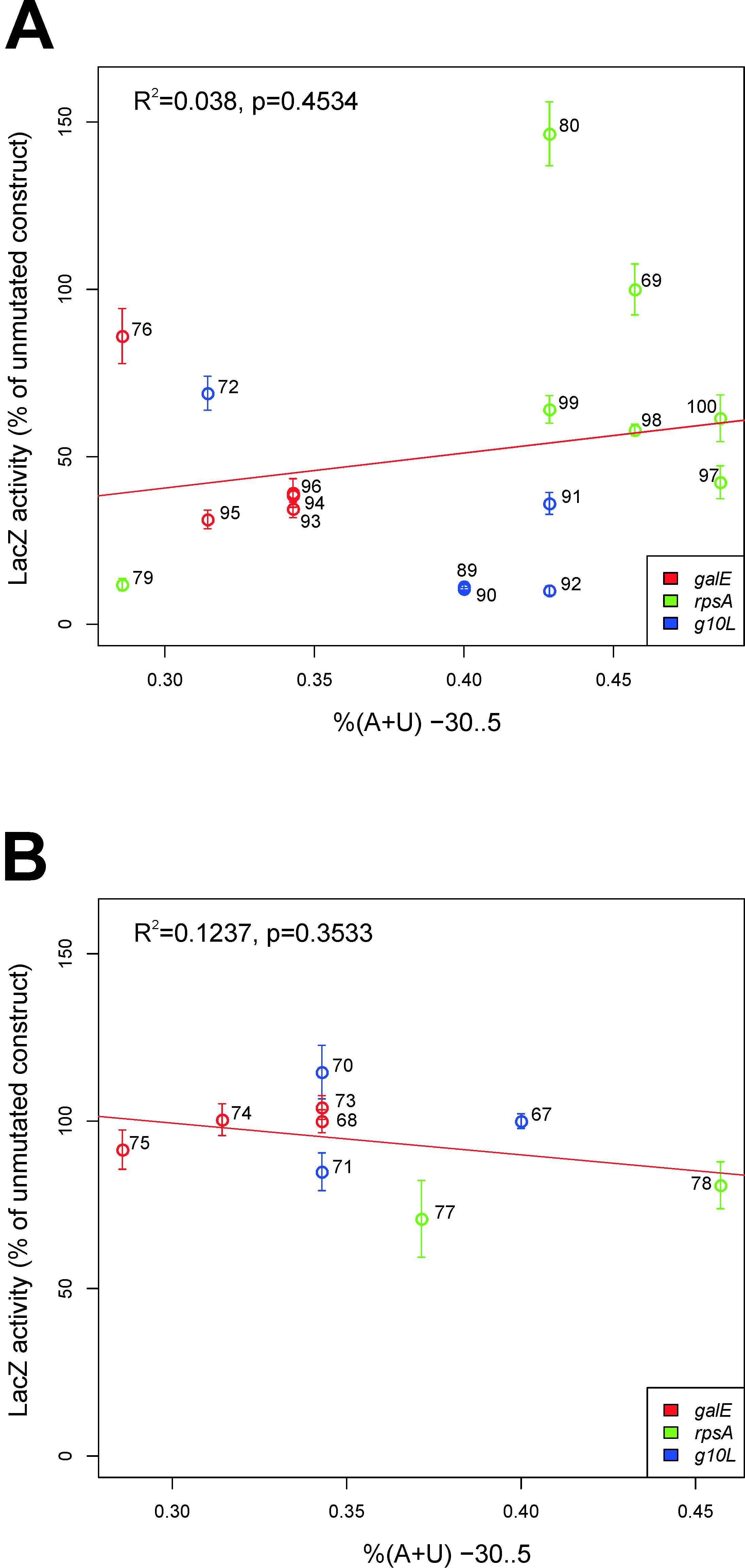

Supplement: Figure S8 — Correlation between AU content in the −30 to −5 region and LacZ activity in reporter gene constructs. Lack of a significant correlation excludes the possibility that the efficiency of translation initiation depends on binding of the ribosomal protein S1 via AU-rich sequences in the 5′ UTR [9], [11]. (A) Correlation between AU content and LacZ activity in constructs without SD sequence. (B) Correlation between AU content and LacZ activity in constructs with SD sequence. (TIF) [file pgen.1002155.s008.tif]

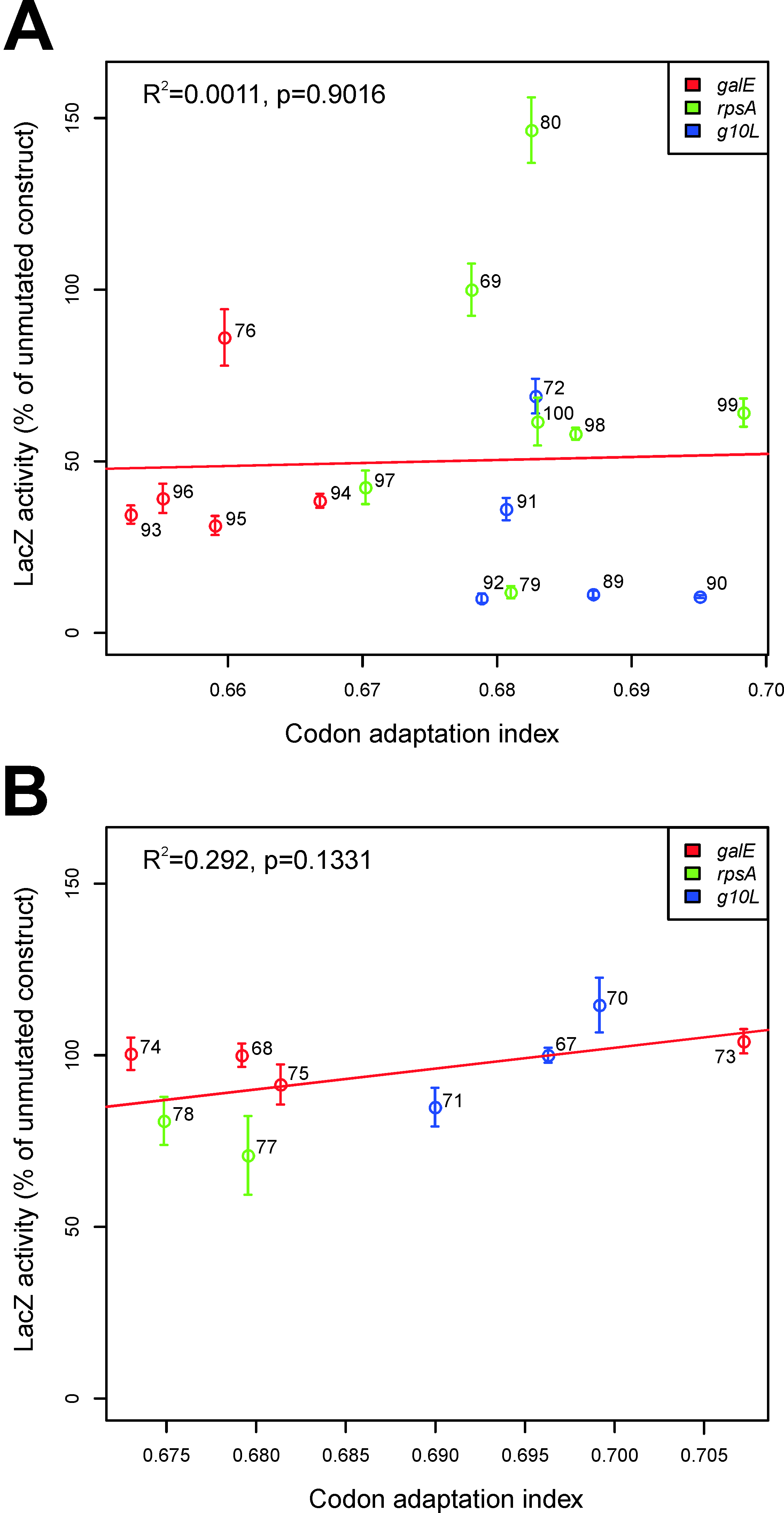

Supplement: Figure S9 — Correlation between codon adaptation index (CAI) and LacZ activity in reporter gene constructs without SD sequence. The Codon Adaptation Index (CAI) was calculated from the relative synonymous codon usage (RSCU) table for Escherichia coli. (A) Correlation between CAI and LacZ activity in constructs without SD. (B) Correlation between CAI and LacZ activity in constructs with SD sequence. (TIF) [file pgen.1002155.s009.tif]

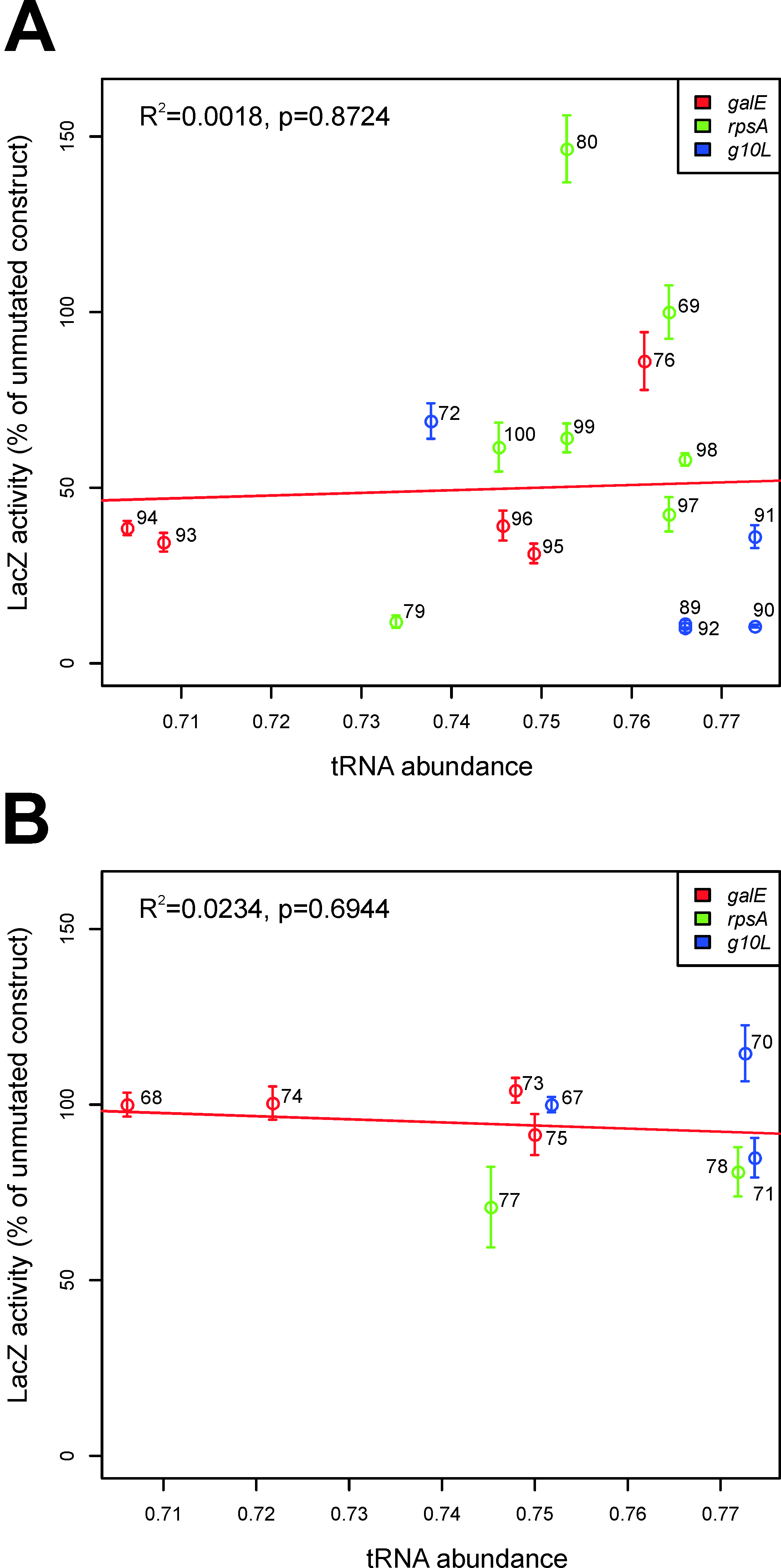

Supplement: Figure S10 — Correlation between tRNA abundance and LacZ activity in reporter gene constructs without SD sequence. Published tRNA abundance measurements [36] were used to calculate the expected expression for each construct. (A) Correlation between tRNA abundance and LacZ activity in constructs without SD. (B) Correlation between tRNA abundance and LacZ activity in constructs with SD sequence. (TIF) [file pgen.1002155.s010.tif]

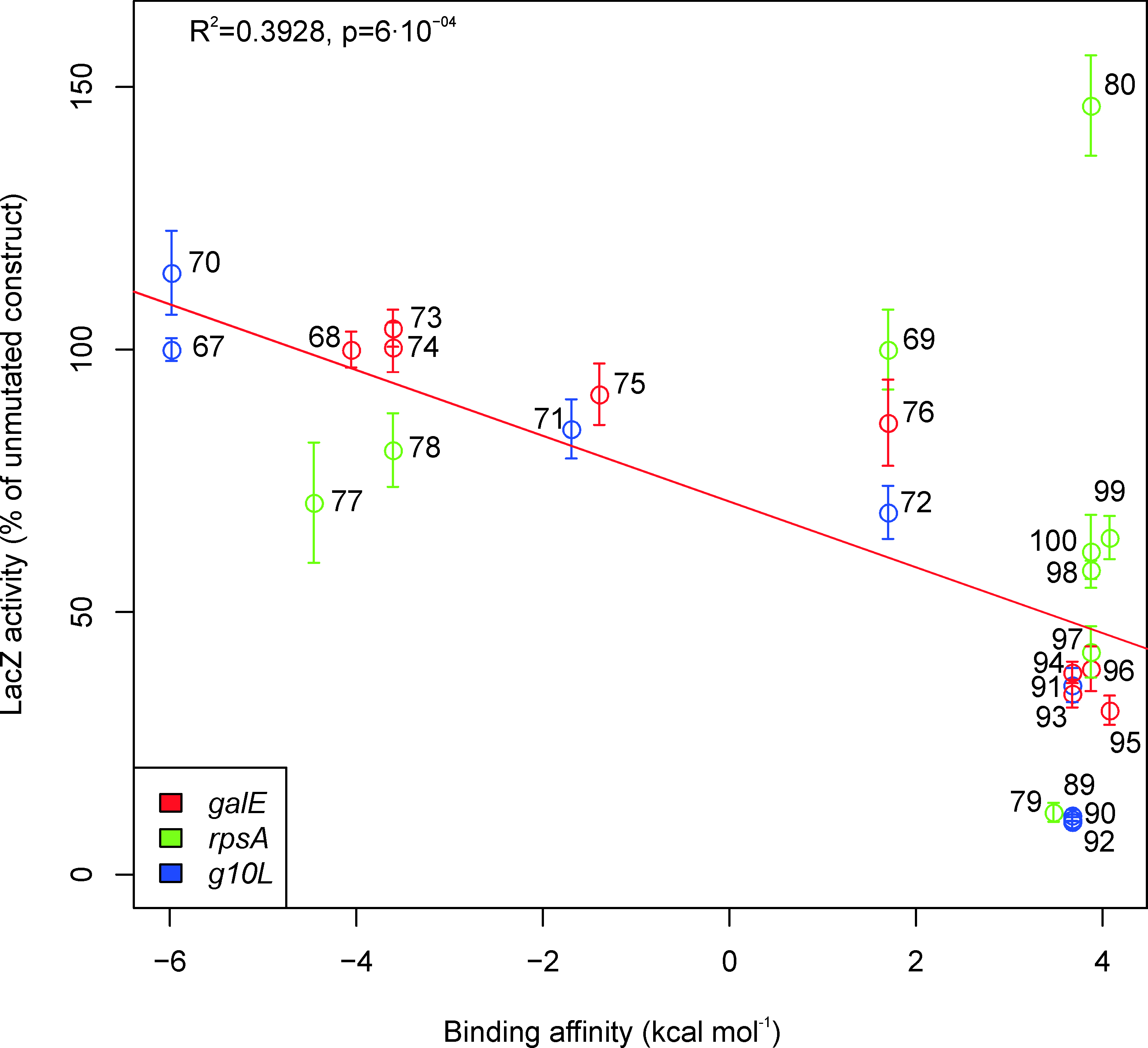

Supplement: Figure S11 — Correlation between LacZ activity and strength of Shine-Dalgarno-type binding between the 5′ UTR (from position -22 to -2) and the ASD (CCUCCU) in the 16S rRNA tail (hybridization energies given in kcal mol−1). There is a significant correlation, because there are many constructs without SD, but also an inaccessible start codon (i. e., constructs that were designed to have low translational activity). (TIF) [file pgen.1002155.s011.tif]
