# Supplementary material for: Local Absence of Secondary Structure Permits Translation of mRNAs that Lack Ribosome-Binding Sites
Source: PLoS Genet. 2011 Jun 23;7(6):e1002155. doi: 10.1371/journal.pgen.1002155 (PMC3121790; doi:10.1371/journal.pgen.1002155)
Supplement: Table S1 — List of oligonucleotides used in this study. Primer numbers correspond to construct numbers. (DOC) [file pgen.1002155.s012.doc]

| PLS67for | TCTCTAGATTACCATTCGCCATTC |
| --- | --- |
| PLS67rev | ACCCATGGCCATGATTACGGATTCAAGATCTCTGGCCGTCGTTTTAC |
| PLS68for | AGAGATCTACCGGTAACCAGAACTCTCATAATTCGCTCCATTAGG |
| PLS68rev | AGGATCCGGTTATTTCATACCATAAGCCTAATGGAGCGAATTATG |
| PLS69for | AGAGATCTTTGAGCAAAAGATTCAGTCATGTTTAATCTTCAGGTTTATATTTAACGTCCACCTGGCTCCATG |
| PLS69rev | AGGATCCCCCCGTTGCAATGGAATGACAGCGGGTATGTTAAACAACCCCATCCGGCATGGAGCCAGGTGGAC |
| PLS70for | AGAGATCTTGAATCCGTAATCATCGCCATAGAAGGCTCTCC |
| PLS70rev | AGGATCCGAAAAAACTAGTCTGCAGAAGGAGAGCCTTCTATGGC |
| PLS71for | AGAGATCTGCTGTCAGTAATCATTGCCATGTTGGCCCGCAGTTCTG |
| PLS71rev | AGGATCCGAAAAAACTAGTCTGCAGAACTGCGGGCCAACATG |
| PLS72for | AGAGATCTCGAATCGGTTATCATGGCCATAGGAAACGAACTTTCTGC |
| PLS72rev | AGGATCCGAAAAAACTAGTCTGCAGAAAGTTCGTTTCCTATG |
| PLS73for | AGAGATCTGCCGGTCACCAGAACACGCATTAATGTCTCCATTAGGC |
| PLS73rev | AGGATCCGGTTATTTCATACCATAAGCCTAATGGAGACATTAATGCG |
| PLS74for | AGAGATCTTCCGGTAACAAGAACACGCATCGCTTACTCCATTAGG |
| PLS74rev | AGGATCCGGTTATTTCATACCATAAGCCTAATGGAGTAAGCGATGC |
| PLS75for | AGAGATCTACCCGTGACAAGCACACGCATAACCCGGGTTATTAGG |
| PLS75rev | AGGATCCGGTTATTTCATACCATAAGCCTAATAACCCGGGTTATGC |
| PLS76for | AGAGATCTTCCGGTCACTAGTACACGCATGCGTTGGACGATTAGG |
| PLS76rev | AGGATCCGGTTATTTCATACCATAAGCCTAATCGTCCAACGCATG |
| PLS77for | AGAGATCTCTGGGCGAAGGACTCAGTCATAGTCGGTCTCCAGGTTTATATTTAACGTCCACCTGGCTCCATG |
| PLS78for | AGAGATCTTTGTGCGAAGCTCTCTGTCATTTGTTATCTCCAGGTTTATATTTAACGTCCACCTGGCTCCATG |
| PLS79for | AGAGATCTTTGGGCAAACGACTCCGTCATAAGACGGCGGGAGGTTTATATTTAACGTCCACCTGGCTCCATG |
| PLS80for | AGAGATCTCTGGGCAAAACTCTCTGTCATTGTAGTATATAAGGTTTATATTTAACGTCCACCTGGCTCCATG |
| PLS89for | AGAGATCTGGAGTCTGTAATCATCGCCATCCTTATATGGGTGG |
| PLS89rev | AGGATCCGAAAAAATAGATTCGCCACCACCCATATAAGGATGG |
| PLS90for | AGAGATCTACTATCAGTAATCATCGCCATACTGGCGGATGAATG |
| PLS90rev | AGGATCCGAAAAAAAGGACAATACCATTCATCCGCCAGTATG |
| PLS91for | AGAGATCTACTGTCAGTAATCATAGCCATTCCGGAATATATTG |
| PLS91rev | AGGATCCGAAAAAAAGAGCACCCATCAATATATTCCGGAATGGC |
| PLS92for | AGAGATCTGGAGTCTGTAATCATAGCCATTCATATATTGCGATGG |
| PLS92rev | AGGATCCGAAAAAAAGAGCACCCCCATCGCAATATATGAATGG |
| PLS93for | AGAGATCTACCAGTAACAAGAACTCTCATTCTTATATGGGCGCG |
| PLS93rev | AGGATCCGGTTATTTCAGGGATATAACCGCGCCCATATAAGAATG |
| PLS94for | AGAGATCTACCAGTAACCAGGACTCTCATCTCGCGGTTATATGTG |
| PLS94rev | AGGATCCGGTTATTTCAGGAATATTCCACATATAACCGCGAGATG |
| PLS95for | AGAGATCTACCGGTCACTAGCACTCTCATTCGTTATATATGGG |
| PLS95rev | AGGATCCGGTTATTTCAGGCTTCAATTTCCCATATATAACGAATGAGAG |
| PLS96for | AGAGATCTGCCCGTAACTAGCACTCGCATTCTTGGGTATATATGG |
| PLS96rev | AGGATCCGGTTATTTCAAGAATTTTCCCATATATACCCAAGAATGCG |
| PLS97for | AGAGATCTTTGAGCAAAGGACTCAGTCATCATTTAATTAAATGATGTTATTTTTCGTCCACCTGGCTCCATG |
| PLS98for | AGAGATCTCTGAGCAAAAGATTCAGTCATCTTTTGGTTGAATATTATTATTCAACGTCCACCTGGCTCCATG |
| PLS99for | AGAGATCTCTGGGCAAAGCTTTCAGTCATCAGTGAAAATTATTATTATGTTTTACGTCCACCTGGCTCCATG |
| PLS100for | AGAGATCTCTGGGCAAAAGACTCAGTCATTTTTATTATATATTGAATTGTTCAGCGTCCACCTGGCTCCATG |
